# Exploring the first possessor bias in children

**DOI:** 10.1371/journal.pone.0209422

**Published:** 2019-01-17

**Authors:** Nicholaus S. Noles, Frank C. Keil

**Affiliations:** 1 Department of Psychological and Brain Sciences, University of Louisville, Louisville, KY, United States of America; 2 Department of Psychology, Yale University, New Haven, CT, United States of America; Consiglio Nazionale delle Ricerche, ITALY

## Abstract

Even very young children are adept at linking property to owners (Gelman, Manczak, & Noles, 2012). However, some studies report that children systematically conserve property with the first possessors (Blake & Harris, 2009; Friedman & Neary, 2008). The present study seeks to integrate these two findings by testing for the presence of a first possessor bias in older children (ages 7–10) using a broader array of property transfers, and by investigating how manipulations of context–from third-person to first-person–yield ownership attributions that are more or less biased. Seven- and 8-year-olds, but not older children, exhibited a first possessor bias when property transfers were presented in a third-person context. This finding suggests that the first possessor bias persists longer in childhood than previously suspected. However, the bias was greatly attenuated or absent when property transfers were presented in a first-person context, rather than a third-person context.

## Introduction

The modern human environment is cluttered with, and constructed of, property, but the links that bind people to property are invisible and abstract. Moreover, property ownership is somewhat fluid. The exchange of something for something, quid pro quo, is a universal human experience that permeates economic and interpersonal interactions. The ubiquity of property, and the frequency with which it changes owners should make tracking the relationships between people and property difficult. However, we do so efficiently in a manner that is largely automatic and effortless. This juxtaposition of complexity and fluency has inspired investigations exploring the emergence and development of concepts of ownership and property in children.

Developing mature concepts of ownership and property go far beyond knowing what is “mine” and not “mine.” Ownership concepts are thought to be early emerging [1, 2, 3, 4] and experienced across cultures [5, 6], which suggests that ownership concepts are a basic part of human cognitive architecture. Most children begin to use possessive language by age two to define property and to defend their possession of objects [7, 8, 9]. Although two-year-olds are notorious for their outlandish ownership claims, they exhibit an impressive capacity for marking and tracking owners [10] and property [11], and they experience the endowment effect (i.e., an increased liking or valuation of personal property relative to other, identical objects) for property given to them [12, 13], just as adults do [14].

By age three, children use the same heuristic that adults use, first possession [15], to determine ownership in ambiguous situations [16]. When presented with explicit ownership cues, preschoolers automatically track property locations, object history, and recall ownership associations [12]. Moreover, much like the tracking of ownership of art through provenances that trace the history of transactions, determining the ownership of any present object requires a sense of its past and how it was embedded in actions between various intentional agents [12, 17, 18]. Children perform these impressive tasks with minimal formal training, seeming to naturally comprehend many facets of ownership despite inconsistent and often contradictory information provided by parents [19]. Thus, notions of ownership are apparently intuitive to even very young children.

An early ease with ownership ideas, however, does not mean that young children understand ownership in the same way as older children and adults. Children commonly violate the rules that govern transfers of property in everyday life. Many parents have had the experience of leaving a store only to discover during the ride home that their child has mysteriously acquired a new toy or a piece of candy. However, although violations of this nature are salient, they are surprisingly rare given the ubiquity of property.

Although young children begin to grasp the normative dimensions of property rights at around age three [20], their ownership attributions are biased in systematic ways. Children often fail to grant the full suite of property rights to owners. For example, children do not believe that owners have the right to destroy their property [21, 22]. Also, children consistently link ownership to object creators [23] and individuals who invest labor into objects [24], and cues such as proximity [16] and control of permission [25] help them to identify owners when other ownership cues are ambiguous. These findings are apparent cross-culturally [6], and adults are also sensitive to similar cues and exhibit similar biases [15, 24, 26, 27], although more nuanced methods and measures are sometimes required to demonstrate these effects in adults.

One consistent finding in the literature is that children exhibit an over-arching bias to preserve property rights with individuals who first possess an entity [16, 28]. Friedman and Neary [16] found that preschoolers exhibit a “first possessor bias,” attributing ownership to an object’s first possessor, and not to subsequent possessors, even when the property was explicitly transferred. The first possessor bias strongly influenced children’s ownership attributions, but Friedman and Neary discovered that three- and four-year-olds did not exhibit the bias when they were presented with a familiar context, such as a birthday party scenario featuring a wrapped gift. Similarly, Blake and Harris [28] also reported that two- and three-year-olds exhibit a powerful first possessor bias that was not present in five-year-olds. These two studies provide converging evidence that preschool aged children’s understanding of property transfers and ownership attributions are powerfully biased until age four or five. However, Kanngiesser and colleagues [5] presented similar scenarios to the Kikuyu people of Kenya, who live in small-scale groups and have little or no personal property as children, and found that they exhibited a similar bias, but with a delay of several years (i.e., Kikuyu children’s judgments were less biased toward first possession at age 5 than children in more property-rich cultures, but the previously reported bias appeared in Kikuyu children by ages 8 or 9.) The present study explores this bias in greater detail by addressing three questions: 1) How persistent is the first possessor bias? 2) Does the first possessor bias influence property transfers beyond giving? 3) What role does context play in children’s ownership attributions?

### How persistent is the first possessor bias?

Prior reports [16, 28] present compelling evidence that children exhibit a first possessor bias. The surface-level conclusion that can and has been drawn from these reports is that children exhibit an early bias that is attenuated or absent by age four or five. However, elements of these two reports leave the scope of the first possessor bias unclear. Friedman and Neary [16] established that this bias was present in children, and they further demonstrated that the effect was only attenuated when the property was described as a “present” and the visual representation of the present was a wrapped gift. Under most other conditions, children determined that the first possessor continued to own the property or responded at chance. Thus, Friedman and Neary defined the conditions sufficient to provoke children to attribute ownership to a new owner, but not the developmental boundaries of the bias. Blake and Harris [28] tested slightly older children, and concluded that children exhibit a “mature understanding of ownership transfer” by age 5. However, their procedure employed a birthday party scenario that explicitly referenced wrapped gifts.

Certainly, these two studies identify a powerful bias and define circumstances under which children overcome it. However, it is not obvious whether the broader claim presented by Blake and Harris, that children understand ownership transfers by age 5, is supported by these findings. An alternative, and more restrictive, interpretation of these findings is that children’s understanding of property transfers is subject to a general bias, unless they are presented with a salient, familiar, and highly scripted context (i.e., a birthday party) in which a familiar script can override their biases. It is also possible that wrapping a gift and attending a party more clearly communicates the gift-giver’s intention to transfer their property. In addition, the developmental start- and end-points of this bias also remain undefined. Blake and Harris [28] set an endpoint at age five, but Kanngisser and colleagues [5] found that this bias emerged later in a culture where children have little personal property. Minimally, this finding suggests that there might be more complexity to defining the scale of the first possessor bias than previously indicated. In order to more fully characterize the breadth of the first possessor bias, the current study focuses on older children, including a group of 7- to 8-year-olds and a group of 9- to 10-year-olds. We believe that prior work has overestimated the maturity of children’s ownership concepts, and thus we predict that the first possessor bias extends beyond age 5, as no prior study of property transfers using these methods demonstrates adult-like intuitions in children without employing references to wrapped gifts and birthday parties.

### Does the first possessor bias influence property transfers beyond giving?

Prior studies focusing specifically on property transfer focused on children’s intuitions under ambiguous circumstances [5, 16], where children were required to decode the disposition of people and property and make ownership attributions, or children’s intuitions about unambiguous legitimate (giving) and illegitimate (stealing) property transfers [28]. The present study does not require children to decode ambiguous circumstances. Instead, we presented children with a broad range of unambiguous scenarios in order to determine whether the first possessor bias is limited to gift-giving scenarios, or if it extends to other categories of property transfer. In particular, this investigation presents children with explicit property transfers (e.g., giving and selling), non-transfers (e.g., lending and borrowing), and property loss in neutral (i.e., non-birthday) scenarios. We predict that evidence of the first possessor bias will be apparent in explicit property transfers, replicating prior findings, and appropriately applied to non-giving scenarios as well.

### What role does context play in children’s ownership attributions?

Friedman & Neary [16] demonstrated that contextual factors powerfully influence children’s ownership attributions. Referencing wrapped presents and birthdays dramatically changed how children determined property ownership. The question remains, however, whether children’s more mature response patterns when presented with birthday party scenarios is attributable to the specific nature of that scripted sequence, which is both highly salient and often coached by parents, or if the shift in children’s responses reflects a broader sensitivity to contextual information. As a test case, we will focus on the shift between experiencing property transfers as a third-party observer, and directly participating in a property transfer. We predict this shift in context will be relevant for two reasons. First, although prior reports may have accurately measured and described children’s behaviors, anecdotally, preschool age children do not seem to be confused when they observe or participate in property transfers. The existence of a robust and influential first possessor bias would be less interesting if children made many obvious mistakes when reasoning about property, but they do not. Second, a transition from an understanding that develops first for the self and is then extended to others is a hallmark of several aspects of child development (e.g., Theory of Mind [29]). Indeed, Gelman, Manczak, and Noles [12] provided evidence for exactly this manner of developmental transition in young children’s tracking of property and ownership. They found that 2- and 3-year-olds marked and tracked property assigned to them more accurately than property assigned to others, but that the tracking of others’ property improved with age. Thus, we predict that children will exhibit a first possessor bias when property transfers are presented to them as third-person vignettes, but that this bias will be attenuated when similarly structured scenarios are contextualized as first-person experiences. We propose that context provides a mechanism by which children’s biased intuitions may appear–to the casual observer–typical and mature.

The literature investigating self-serving biases provides a broader context for these findings [30]. Self-serving biases reflect the human tendency to shift judgments and intuitions into their own favor, and such biases provide a natural counter-weight to conservative biases, such as the first possessor bias. This formulation positions the first possessor bias as one of a set of biases operating when children make ownership attributions, rather than assuming that children’s intuitions are simply biased relative to a normative interpretation of judging the outcomes of property transfers. The manipulation of context employed in the present study provides a mechanism for discriminating between these two competing interpretations.

### The current study

The goal of the present study is to explore attributions of ownership in older children across a variety of different property transfers presented in neutral contexts. In addition, we will explore the influence of context on children’s intuitions in an effort to elucidate how biases factor into children’s ownership attributions. We elected to probe children’s explicit understanding of ownership for two reasons. First, this was the method employed in prior studies [16, 28], and our intention was to replicate and extend that work. Second, although there are more nuanced methods for probing children’s intuitions, such as measuring reaction time or object orientation [26], these methods are not well-suited to testing children (e.g., reaction times are much more variable in children than adults), and they are not well-suited for use in children’s educational environments. Thus, the current study replicates and extends prior work while providing additional information critical to evaluating claims about the first possessor bias and further clarifying its nature and scope.

## Experiment 1

In Experiment 1, participants were presented with 14 scenarios depicting property transfers in a third-person observer context. The scenarios included depictions of property transfers falling into three categories. The first category, “Transfers,” included unambiguous property transfers such as giving, selling, and “ungiving,” which describes circumstances where an object is intentionally transferred from one actor to another and then the first possessor expresses a wish for the object to be returned. We also presented participants with “Losses,” events such as discarding or losing an item, as well as theft. Finally, we presented participants with “Non-transfers,” events that were schematically similar to property transfers, but that did not actually involve the transfer of property rights, including borrowing, the passage of time, changes in proximity, and a case we refer to as “lingering,” which involves a non-transfer followed by the removal of the first possessor (see S1 Appendix, item 8). Critically, we avoided using birthday scenarios or language related to these scenarios (e.g., presents, gifts, etc.).

These stimuli were presented to adults and children in order to identify developmental trends in ownership concepts across different Transfer-types and to provide a baseline context for comparison with the first-person actor context employed in Experiment 2. The earliest adult-like responses recorded in previous studies were provided by five-year-olds, but other studies indicate that eight-year-olds sometimes made errors in attributing property rights [22] and identifying owners [10]. Given these data, and the failure of younger children to make adult-like attributions in prior studies of property transfers, the current study focuses on children ages 7 to 10. Also, previous investigations examining property transfers employed complex scenarios involving multiple actors and shifting relationships between actors and property. Testing slightly older children increases the probability that their responses reflect their developing ownership concepts, and not their cognitive limitations.

### Methods

The data included in this report were collected ethically with informed consent and without coercion. This study was reviewed and approved by the Internal Review Board at Yale University. For child participants, written consent was provided by a parent or guardian, and the child’s assent was obtained before testing. For adult participants, this project was given an “exempt” status because it represented minimal risk and no personal or protected health information was collected. Thus, adult participants were provided with information about the study (as would appear on an informed consent form), but they were only required to provide verbal consent in order to participate.

#### Participants

Twenty 7- and 8-year-olds (*M*_*age*_ = 8;5, 12 female) and twenty 9- and 10-year-olds (*M*_*age*_ = 10;4, 6 female) were recruited from schools in rural Alabama and suburban Florida. Child participants were primarily Caucasian-Americans from middle-class backgrounds, as estimated from usage of school lunch programs. Signed informed consent forms were completed by a parent or guardian for each child, and each child verbally assented to testing before leaving their classroom. Adults (*N* = 20, 11 female) were recruited from a sample of university students completing experiments for course credit. This sample was drawn from a primarily Caucasian-American population of Yale University undergraduates. For adults, this project was designated as “Exempt” by the Internal Review Board of Yale University because a) it was determined to pose the minimal possible risk to participants and b) it did not require the collection or storage of any identifying information or protected health information. This project was thus exempted from typical consent requirements for adults. Each adult was provided with a sheet providing information about the study, as one might see on an informed consent form, but they were only required to verbally consent to the study in order to participate. This exemption limited the data that we could collect on each adult, thus we know that all adults were over the age of 18, but the exempt status of this work prevented the collection of any additional demographic data.

#### Stimuli & procedure

The stimuli used in Experiment 1 consisted of neutral, third-person scenarios falling into one of three categories, Transfers, Losses, or Non-transfers. At the end of each scenario, children were asked to identify who owned each piece of property. Many investigations either explicitly or implicitly avoid the word “own,” using possessive language or phrases like “belongs to,” to probe children’s understanding of ownership in the absence of their explicit understanding of the word “own.” A preliminary study with 18 children (*M*_*age*_ = 6.39, 10 female) found that children overwhelmingly treated the terms “own” and “belongs to” as synonymous. Thus, we elected to use the word “own” in our materials.

Most scenarios involved straightforward property transfers or common non-transfers involving two individuals (see S1 Appendix). However, several scenarios were manipulated and presented more than once in order to determine whether value or justification played a critical role in inferences of ownership. It is possible that children’s conservatism in inferring property transfers may be influenced by property value. For example, the influence of the first possessor bias may be related to the value of the to-be-transferred property, rising or falling with the value of depicted items. Thus, two scenarios were concerned with losing and finding property, including events where both low-value objects (e.g., a pen) and high-value objects (e.g., a diamond) were lost (children’s specific value judgments vary wildly, but their relative judgments are typically accurate [18]). Similarly, two ungiving scenarios depicted property of either high or low value. We also manipulated two ungiving scenarios with justifications for ungiving that were more or less equitable (e.g., see S1 Appendix, items 6 and 7). This manipulation therefore explores whether the quality of the justifications for ungiving influences children’s intuitions about the permanence of property transfers, perhaps leading children to endorse ungiving “for a good cause” while rejecting ungiving that seems unfair or spiteful.

Adults received a pen and paper questionnaire, and children interacted with an experimenter who read the scenarios aloud and pointed to pictures representing the actors and target items. The pictures consisted of black and white line drawings of the named objects and line drawings of two adult males or females. In order to minimize proximity effects [16], all property was depicted equidistant from each possible owner. All trials were randomized in order to determine the initial order of presentation for the items, and half of the participants received the items in reverse order.

We collected data from adult participants first and we did not present children with any item that the adult sample responded to ambiguously (i.e., chance responding as calculated using the binomial theorem). Our goal was to present children with only those items that yielded clear and systematic intuitions in adults. This resulted in the exclusion of the “accidental giving” scenario from the materials presented to children. Participants were instructed to listen to each scenario carefully, and then asked to select the owner of each target item. The experimenter recorded the children’s responses.

### Results & discussion

Composite scores representing selection of first possessors were calculated by collapsing responses by Transfer-type into a score from zero to one, representing the proportion of trials for each trial type where they selected a first possessor as the owner of the target property in each vignette (data available at Databrary.org). An omnibus two-way analysis of variance (ANOVA) with Age as a between-subjects factor and Transfer-type as a repeated measure revealed significant main effects for both Age (*F*(2, 57) = 26.60, *p* < .001, η^2^ = .483) and Transfer-type (*F*(2, 56) = 138.12, *p <* .001, η^2^ = .831), as well as a significant Age X Transfer-type interaction (*F*(4, 114) = 12.69, *p <* .001, η^2^ = .308). In order to more closely examine these effects, we employed a Kruskal-Wallis test for several independent samples to identify items that contributed to these effects. As shown in Table 1, children and adults demonstrated a large degree of agreement when making ownership attributions following Losses and Non-transfers.

**Table 1:** First possessor endorsement by age and transfer-type in experiment 1.

		% endorsing first possessor			
	Item	Ages 7 & 8	Ages 9 & 10	Adults	*χ* ^*2*^ (2, *N* = 60)
	time	95	100	100	2.00, *p* = .368
Non-transfer	proximity	90	85	100	3.00, *p* = .223
	borrow	95	80	100	5.58, *p* = .061
	linger	90	100	100	4.07, *p* = .131
					
	give	65	5	5	25.17, *p* < .001
	sell	45	5	0	17.23, *p* < .001
	ungive +value	65	10	0	25.70, *p* < .001
Transfer	ungive -value	70	10	15	20.15, *p* < .001
	ungive +explanation	65	15	0	23.30, *p* < .001
	ungive -explanation	60	05	10	19.40, *p* < .001
	accidental giving	-	-	30	-
	loss+value	90	75	85	1.65, *p* = .438
Loss	loss-value	95	75	75	3.50, *p* = .174
	steal	95	90	95	0.53, *p* = .768
	discard	75	35	5	20.52, *p* < .001

All percentages are based on the percentage of each population that endorsed the first possessor as the owner when queried after each scenario. For ungiving and loss items, “+value” and “-value” represent manipulations in high versus low value, respectively. Likewise, “+explanation” and “-explanation” represent ungiving justified with either more or less equitable explanations. Data is missing from the accidental giving row because children did not receive this item.

However, 7- to 8-eight-year-olds systematically differed from 9- and 10-year-olds and adults in their conclusions regarding intentional transfers of property, including all Transfer items (intentional transfers to another actor) and the discard item (a “loss,” but nevertheless an intentional transfer away from the owner). Specifically, children under 9 commonly inferred that first possessors maintained ownership of property, even after they unambiguously transferred the property to another person. In addition, we found no evidence that manipulating the value of items involved in ungiving and property loss or the equitability of justifications for ungiving significantly influenced ownership attributions.

Using a wider variety of property transfers than employed in prior studies, Experiment 1 reveals that the first possessor bias influences ownership attributions among children age 7 and 8, but not 9 and 10. This finding replicates prior studies with children from property-dense backgrounds, and it contextualizes the process of overcoming the first possessor bias. Initially, the first possessor bias is only attenuated in the context of a birthday party, but the bias is attenuated in 9- and 10-year-olds even when this schema is absent. These findings also align more generally with findings presented by Kim and Kalish [22], who report errors in reasoning about property rights in children until at least age 10. These findings, considered in concert with the data reported above, indicate that conservative biases regarding property broadly influence children’s intuitions and ownership attributions until approximately age 10.

## Experiment 2

Experiment 1 demonstrates that the first possessor bias persists much longer into development than previously thought. However, this result seems to conflict with anecdotal evidence that children *do* often understand property exchanges, and even reason about them effectively, at a very young age. Certainly, the literature contains many examples of children’s precocious understanding of ownership and property. For example, Gelman and colleagues [12] provide evidence that 3-year-olds mark, track, and remember property assignments without explicit instruction even though doing so requires attention and the spatiotemporal tracking of individual objects over time. Furthermore, if their ability to track property is defeated, children will spontaneously search for other evidence (e.g., marks or other physical or visual cues) to assist them in linking people to property [31]

Critically, there are important contextual differences between studies and situations where children reason effectively about ownership and where they do not. Specifically, children track and remember to whom property belongs [1, 12, 32] when they are integrated into the web of interpersonal connections with the property. Put more simply, when they are a first-person observer or participant in manipulations of property and ownership, children’s intuitions are not biased in the same manner as when they are presented with third-person vignettes. Thus, in Experiment 2 we shifted participants from a third-person observer context to a first-person actor in property transfer scenarios.

There are at least two possible elements of first-person interactions that may help children to make more adult-like ownership attributions about property transfers. First, the first-person receipt of property from another person mirrors children’s everyday experiences with property transfers, which largely consists of receiving property from adults. Second, receiving property is literally a “personal gain,” and the history of psychology is filled with evidence of self-serving biases, including many scenarios wherein individuals present intuitions that differ across contexts [33]. These contextual shifts often result in participants resolving ambiguous situations in a manner that enhances personal gains and attributes positive outcomes to personal factors [30]. In combination, these two factors may provoke adult-like ownership attributions in children, and provide a mechanism by which their attitudes toward property may appear to be reasonable and complete in everyday life, but powerfully biased when recorded in research studies. We predict that the first possessor bias will be significantly attenuated when property transfers are presented in a first-person context.

### Methods

#### Participants

Eighteen 7- and 8-year-olds (9 male, *M*_*age*_ = 7;6) and twenty 9- and 10-year-olds (11 male, *M*_*age*_ = 10;2), and twenty adults were recruited from the same populations as Experiment 1.

#### Stimuli & procedure

Stimuli and procedures in Experiment 2 were schematically similar to those used in Experiment 1. Rather than presenting children with depictions of owners and objects, experimenters presented children with unfamiliar, low-value objects. Children reasoned effectively about such objects in several prior studies by Gelman and colleagues [12, 18]. Each object was randomly paired with one of the items from Experiment 1 and presented as a transaction (see S1 Appendix for item-object pairings). For example:

Experimenter: “I let you borrow this [*indicates felt ball*] for the weekend. [*The object is placed equidistantly between participant and experimenter*.] Who owns this [*indicates object*]?”

Children were asked to listen carefully because the experimenter would be asking them questions. The experimenter then presented nine scenarios from Experiment 1 (marked with asterisks in the S1 Appendix). Because there were no strong effects of our manipulations of ungiving and property loss in Experiment 1, we only presented participants with one instance of each in Experiment 2. We also removed an additional item (#8 in the S1 Appendix) in order to reduce the overall length of the experimental session. We randomized and counterbalanced the remaining items as in Experiment 1. Participants were always framed as the recipients or second actor in each scenario, and they were asked who owned the target object at the end of each trial. Participants indicated that the item either belonged to them or to the experimenter. Children were able to mark and track similar objects in studies reported by Gelman and colleagues [12], demonstrating that such interactions were sufficiently salient to provoke ownership-related biases (e.g., the endowment effect) and object tracking. Thus, we were confident that participants could understand and evaluate the relevant elements of these simple interactions. This task was, in terms of complexity, much easier than tasks completed by 2-year-olds in prior studies. The experimenter recorded participants’ responses for each trial and put each object away before beginning the next trial. Children and adults experienced identical procedures.

### Results & discussion

As in Experiment 1, we collapsed participants’ responses into composite scores and analyzed them with a repeated measures ANOVA using Transfer-type as a within-subjects factor and Age as a between-subjects factor. This test revealed significant main effects for both Age (*F*(2, 55) = 5.11, *p* = .009, η^2^ = .157) and Transfer-type (*F*(2, 54) = 55.85, *p <* .001, η^2^ = .674), as well as a significant Age X Transfer-type interaction (*F*(4, 110) = 5.57, *p <* .001, η^2^ = .169). We employed a Kruskal-Wallis test for several independent samples to identify items that contributed to these effects. The pattern of responses to Non-transfers was very similar to the pattern demonstrated in Experiment 1 (see Table 2).

**Table 2:** First possessor endorsement by age and transfer-type in experiment 2.

		% endorsing first possessor			
	Item	Eight-year-olds	Ten-year-olds	Adults	*χ* ^*2*^ (2, *N* = 58)
	time	100	100	100	0.00, *p* = 1.000
Non-transfer	proximity	94	100	95	1.08, *p* = .583
	borrow	83	95	100	4.20, *p* = .123
					
	give	0	0	0	0.00, *p* = 1.000
Transfer	sell	0	0	0	0.00, *p* = 1.000
	ungive	67	20	15	13.50, *p* = .001
	loss	78	75	45	5.62, *p* = .060
Loss	steal	56	65	65	0.46, *p* = .794
	discard	50	20	10	8.31, *p* = .016

All percentages are based on the percentage of each sample that endorsed the first possessor (i.e., the experimenter) as the owner when queried after each item.

However, in contrast to Experiment 1 and prior studies of property transfers, 7- and 8-year-olds demonstrated no first possessor bias relative to adults when presented with straightforward property transfers, such as giving and selling. Younger children continued to demonstrate a significant first possessor bias relative to adults and older children when presented with ungiving and discarding scenarios, suggesting that aspects of these particular property transfers (e.g., the lack of a new owner in the discard scenario or the mismatch between actions and intentions in the ungiving scenario) may encourage them to respond more conservatively.

Perhaps the most surprising findings in Experiment 2 were the responses that participants provided for Loss items. In Experiment 1, participants demonstrated a strong predisposition to state that an unintentional loss or a theft did not result in a change of ownership, mirroring findings from other investigations with respect to stealing [16, 22, 28]. The only exception to this pattern was the discard item, which depicted an intentional loss. In Experiment 2, *all age groups* demonstrated attenuated endorsement of the first possessor with respect to stealing, and adults demonstrated attenuated endorsement of first possessor for the unintentional loss item (i.e., finding) as well, which contributed to the marginal effect of Age for this item. In other words, they preserved ownership with first possessors when observing an actor finding property, but when *they* were the finder, they more often judged that the found property belonged to them. Taken together, these findings suggest that context powerfully influences intuitions about property transfers in both children and adults.

## General discussion

Experiment 1 shows that 7- to 8-year-old children differ from adults and older children in their ownership attributions. Specifically, they demonstrate a first possessor bias, consistently attributing ownership to first possessors, even when property transfers are relatively straightforward (e.g., intentionally giving away or selling property). This result replicates previous findings [16, 28] and expands upon those studies, suggesting that the first possessor bias influences a wider swath of property transfers than previously demonstrated, and that children’s ownership attributions are affected by this bias for longer than previously reported.

Whereas Experiment 1 provides evidence that the first possessor bias is more robust than previously specified, Experiment 2 provides an explanation for the mismatch between intuitions that children *do* understand property transfers early in development [12], and findings that children’s intuitions about property transfers are fundamentally biased. Specifically, manipulating the presentation context (i.e., presenting transfers in a first-person context) resulted in children generating adult-like ownership attributions for typical property transfers such as giving and selling. Taken together, these results indicate that concepts of ownership and property continue to change and develop well over a relatively long timescale, despite the fact that they appear early in development. These experiments also replicate previous findings [16, 28], and provide a means for integrating findings suggesting that children reason very maturely at a very young age [12] with those where systematically biased reasoning is observed in older children [22].

Where does the first possessor bias come from? Kanngiesser and colleagues [5] provide compelling evidence that exposure to, and perhaps ownership of, private property is important for developing the first possessor bias, and the data presented above–in conjunction with prior studies [16, 28]–indicate that time and experience are required in order to overcome this bias. Thus, both exposure to and experience with property must play an important role in the development of ownership concepts. However, context effects also obviously influence children’s judgments.

We often consider the first possessor bias as an error, a problem to be solved, but it may also be construed as an adaptive approach to thinking about property. Young children live in a conditional property environment where their rights are inconsistent and malleable–mothers, teachers, peers, and other adults can intervene on their property rights. Indeed, the information that children receive from their parents about ownership, possession, and property is inconsistent (e.g., parents inconsistently reference ownership, possession, prosociality, and fairness when arbitrating property disputes between siblings [19]). Thus, it is possible that young children are less adept at reasoning about property transfers because these events are more ambiguous, and more likely to be intervened upon, than non-transfer scenarios.

Given these circumstances, maintaining strong bonds between owners and their property may be a more functional approach for young children than reasoning in a more adult-like and “accurate” manner. Moreover, violations of property rights are serious social infractions, and setting high thresholds for detecting property transfers may effectively prevent inferences of property transfers in the presence of weak indicators of ownership. For example, it would prevent children from confusing borrowers or possessors with actual owners, a competency Ross [19] observed in children as young as four.

Biases that conserve property with owners may help children to avoid costly errors (e.g., accidentally stealing), but they also might preclude children from detecting property transfers in *their direction*. This outcome would be maladaptive and, moreover, it would conflict with common intuitions that children *do* understand that they own property that they receive. However, in situations where property is transferred into a child’s possession, self-serving biases may play an especially adaptive role. Self-serving biases are sufficiently strong that–in the current study–simply framing a property transfer as a transfer in their direction caused both children *and adults* to infer property transfers where they may have perceived none as a simple observer.

The relative power difference between giver and receiver may also be a factor to consider. In Experiment 1, property was transferred–or not–between two adult actors, but in Experiment 2 an adult experimenter dictated property Transfers, Non-transfers, and Losses to a child participant. It is possible that this asymmetry biased children’s responses, resulting in an attenuation of the first possessor bias. However, the direction of such a bias would likely be in favor of the adult retaining property ownership. In the current study, children’s intuitions about Non-transfers did not appear to be biased in this way, and their judgments about losses appeared to favor themselves (the child) and not the adult experimenter. Children generally favored the first possessor (the powerful adult) less often overall across Transfer-types. Thus, their shift in response patterns is not likely attributable to power differential between the adult experimenter and the child participant.

Although our results indicate an attenuation of the first possessor bias during the early school years, the mechanism that yields this attenuation remains mysterious. Certainly, time and exposure to property matter a great deal, as demonstrated by Kanngiesser and colleagues [5], but do 9-year-old Kikuyu children need 4 years of experience to overcome the first possessor bias, as 5-year-olds in a property-dense environment do, or does their relatively advanced level of cognitive development shorten this process? More generally, do self-serving biases attenuate children’s conservative biases, or do self-serving biases and the first possessor bias operate simultaneously but unequally (i.e., the self-serving bias overpowers the first possessor bias in some circumstances, but not others)? Finally, it is consistent to discuss the reported results in terms of the first possessor bias, but these data also might be reasonably contextualized as being attributable to a more general “first owner” bias. Indeed, prior work [16, 28] sometimes treats first possession as separable from or contiguous with ownership. Possession and ownership often are contiguous, but the mechanism for these effects are ambiguous in some cases. When possession is the only mechanism for determining ownership, it is clear that children are biased in favor of first possessors (i.e., first possession yields ownership). However, in scenarios where first possession and ownership are contiguous, these biases may result from ownership or first possession. Thus, an important goal for future research is to tease apart these related mechanisms. Exploring the relationship between context and children’s conservative biases is an important future step for understanding children’s social-cognitive development and the development of ownership concepts.

## Supporting information

S1 Appendix Test items. (DOCX)
