# Supplementary material for: Exploring the first possessor bias in children
Source: PLoS One. 2019 Jan 17;14(1):e0209422. doi: 10.1371/journal.pone.0209422 (PMC6336382; doi:10.1371/journal.pone.0209422)
Supplement: S1 Appendix — (DOCX) [file pone.0209422.s001.docx]

**S1 Appendix: Test Items**

1. Richard puts a baseball bat in his trash. Steven finds the bat in the trash as he is jogging and picks it up. Who owns the bat? *wall hook
2. Jane and Melissa share a room. Jane got a bracelet for her birthday and Melissa got a comb. Jane put the bracelet on her nightstand and never wore it for 20 years. Who owns the bracelet? *wall anchor
3. Mike and Bobby share a room. Mike got a book for his birthday. Mike put the book in his closet, and then he traveled all the way to England on vacation while Bobby stayed home. Who owns the book? *wooden peg
4. Peter accidentally dropped a pen on the ground while he was taking a walk. Later, Quinton finds the pen and picks it up. Who owns the pen? *hollow bead
5. John lets his friend Horatio borrow his new skateboard for the weekend. Who owns the skateboard? *felt ball
6. Earl gave his friend Stanley a soccer ball. The next week, Earl decides that he wants the soccer ball back because he thinks that it looks nice in the corner of his bedroom. Who owns the soccer ball?
7. Steven gave his friend Joe a hockey stick. The next week, Steven decides that he wants the hockey stick back because wants to play in a championship hockey game and all of his sticks are broken. Who owns the hockey stick?
8. Ryan lets Terry borrow his bike. The next day, Ryan moves away to Australia and his mother tells everyone that he will never come back. Terry wants to keep the bike, but Sal, another friend of Ryan’s, wants the bike just as much as Terry. Ryan has moved away and both Terry and Sal want his old bike. Who owns the bike?
9. Marshall gives a book to Nolan. Who owns the book? *wire ball
10. Carl gave his friend Billy a notebook. The next week, Carl decides that he wants the notebook back. Who owns the notebook? *foam triangle
11. Timmy has a gold nugget, but he is very weak. Brent takes the nugget from Timmy. Timmy wants the nugget back, but he cannot make Brent return it. Who owns the nugget? *wooden cup
12. Hector has a nice watch. Jason offers Hector fifty dollars for the watch. Hector takes the money and puts the watch in Jason’s hand. Who owns the watch? *plastic square
13. Stacy gave her friend Bijou a very expensive gold necklace. The next week, Stacy decides that she wants the necklace back. Who owns the necklace?
14. Edward accidentally dropped a large valuable diamond on the ground in an airport. Later, Larry finds the diamond and picks it up. Who owns the diamond?
15. Hernando asks Ken for some money. Ken wants to give Hernando five dollars, but he accidentally puts a twenty-dollar bill in Hernando’s hand. Who owns the twenty-dollar bill?

Items used in Experiment 2 are marked with asterisks followed by object names. Participants in Experiment 2 received simplified versions of these test items accompanied by ambiguous objects. The object listed after the asterisk was always paired with the marked test item.
